# Evaluation of Oral Robenacoxib for the Treatment of Postoperative Pain and Inflammation in Cats: Results of a Randomized Clinical Trial

**DOI:** 10.5402/2012/794148

**Published:** 2012-07-01

**Authors:** Stephen King, Elizabeth S. Roberts, Linda M. Roycroft, Jonathan N. King

**Affiliations:** ^1^New Product Development, Novartis Animal Health Inc., Greensboro, NC 27408, USA; ^2^Clinical Development, Novartis Animal Inc., 4058 Basel, Switzerland

## Abstract

The efficacy and safety of robenacoxib were assessed for the control of postoperative pain and inflammation in cats. The study was a multicenter, prospective, randomized, blinded, and parallel group clinical trial. A total of 249 client-owned cats scheduled for forelimb onychectomy plus either ovariohysterectomy or castration surgeries were included. All cats received butorphanol prior to anesthesia and forelimb four-point regional nerve blocks with bupivacaine after induction of general anesthesia. Cats were randomized to receive daily oral tablet robenacoxib, at a mean (range) dosage of 1.84 (1.03–2.40) mg/kg (*n* = 167), or placebo (*n* = 82), once prior to surgery and for two days postoperatively. Significantly (*P* < 0.05) fewer robenacoxib cats received additional analgesia rescue therapy (16.5%) than placebo cats (46.3%). Pain elicited on palpation of the soft tissue incision site, behavior following social interaction, and posture assessed during the first 8 hours after extubation were significantly (*P* < 0.05) improved in cats receiving robenacoxib. Frequency of reported adverse clinical signs, hematology, serum chemistry and urinalysis variables, and body weight changes weresimilar between groups. In conclusion, robenacoxib was effective and well tolerated in the control of postoperative pain and inflammation in cats undergoing onychectomy with ovariohysterectomy or castration.

## 1. Introduction

Pain management has been increasingly recognized as the standard of practice for all types of surgeries in cats including routine elective surgeries, such as, onychectomy, spays and neuters [1–5]. In addition to promoting the welfare of the patient, controlling postoperative pain and inflammation facilitates the healing process and helps avoid the development of chronic pain [3].

The opioids and nonsteroidal anti-inflammatory drugs (NSAIDs) are the classes of analgesics used most frequently for controlling pain in the immediate postoperative period, since they have sufficient potency and duration of action [1, 4]. However, the number of NSAIDs licensed for use in cats is very limited, probably due to the relatively poor safety profile of several NSAIDs in this species [4]. In the USA, only one NSAID, meloxicam, is registered for postoperative pain and inflammation in cats, as a single injectable dose to be administered preoperatively. Repeated administration of meloxicam in cats is not recommended by the Food and Drug Administration-Center for Veterinary Medicine (FDA-CVM, http://www.fda.org/). Therefore there is a need for additional treatments for the control of postoperative pain in cats.

Robenacoxib is a NSAID of the coxib group recently introduced into canine and feline medicine. It possesses analgesic, anti-inflammatory and antipyretic properties [5, 6], and has a wide safety margin in healthy cats which is attributed to its combination of high specificity for the cyclooxygenase (COX)-2 enzyme plus selective distribution and persistence at sites of inflammation [7].

This study was undertaken to assess the efficacy and tolerability of robenacoxib in the control of postoperative pain and inflammation associated with onychectomy/ovariohysterectomy or onychectomy/castration surgeries in cats.

## 2. Materials and Methods

The study was a multicenter, prospective, randomized, blinded, placebo-controlled, and parallel group clinical trial. The study was designed with the primary objective to support registration of robenacoxib by the FDA-CVM. The protocol was reviewed and approved by the FDA-CVM and the Novartis Animal Health Institutional Animal Care and Use Committee. The study was conducted in accordance with Good Clinical Practice (International Cooperation on Harmonisation of Technical Requirements for Registration of Veterinary Medicinal Products, GL9 on Good Clinical Practice, Final Guidance updated 8 June 2011; http://www.fda.gov/). All owners provided written consent before their cats entered the study.

### 2.1. Selection Criteria

Inclusion criteria comprised clinically normal intact cats ≥6 months of age, weighing between 2.5 and 12 kg, presented to the clinic to have a forelimb onychectomy in addition to reproductive sterilization (castration or ovariohysterectomy).

Exclusion criteria included pregnancy; uncontrolled endocrine or systemic disorders (cats requiring treatment for diabetes mellitus or hyperthyroidism had to be stabilized for at least 28 days prior to inclusion); documented or suspected concurrent disease involving the circulatory or coagulation systems, distal limbs, gastrointestinal tract, integument, kidney or liver; other surgery or pain medication in the two weeks prior to inclusion; procedure or presence of chronic pain that would interfere with accurate assessment of pain; alternative forms of pain relief, such as, chiropractic manipulation, acupuncture, acupressure, clinical therapy (e.g., hydrotherapy) within 30 days of inclusion; topical or systemic anti-inflammatory products within 14 days, short-acting (systemic or local) corticosteroids within 30 days or long-acting corticosteroids within 60 days of inclusion into the study; aggressive, fractious, or nervous cats.

Cats could be withdrawn from the study at any time at the discretion of the investigator or owner.

### 2.2. Anesthetic and Analgesia Protocol

With the exception of xylazine or medetomidine (which have analgesic properties), any anesthetic regimen was allowed. All cats were adequately hydrated prior to and during surgery.

To provide an acceptable minimum level of pain control, all cats received 0.4 mg/kg butorphanol subcutaneously as an anesthetic premedication. Butorphanol is registered in the USA in cats undergoing onychectomy with proven efficacy [8]. Once fully anesthetized, the cats received forelimb 4-point regional nerve blocks under aseptic conditions to achieve local anesthesia of the median nerve, palmar branch of the ulnar nerve, and the dorsal digital nerves I to V. The local anesthetic used was 0.5% bupivacaine with the total dosage for both paws not exceeding 5.0 mg/kg [9]. Bupivacaine was selected for its relatively long duration of action, reported to be over 1 hour in cats [10].

Investigators were instructed to administer at any time if needed additional butorphanol or any other product (except other NSAIDs) to the cats as “rescue therapy” for pain control.

Additional analgesic drugs, synthetic feline facial pheromone, corticosteroids, alpha_2_-adrenoceptor agonists, and alternative forms of pain relief (e.g., chiropractic manipulation, or acupuncture) were prohibited.

### 2.3. Surgical Procedures

Onychectomy was performed on both forelimbs, using one of three procedures: guillotine-type nail trimmer, laser scapel, and surgical. Ovariohysterectomy was performed through a standard ventral midline incision; a flank incision was not permitted [11]. Castration was performed via the standard scrotal approach.

### 2.4. Treatment Groups

In addition to butorphanol and regional nerve blocks, cats received either robenacoxib (treated group) or placebo (control group) once daily for three days. The treated group received a minimum dosage of 1.0 mg/kg (range 1.0–2.4 mg/kg) dosed as a 6 mg robenacoxib tablet (Onsior tablets, Novartis Santé Animale, Huningue, France). Control cats received identically formulated placebo tablets without the active ingredient. All dosages were calculated from the preanesthetic body weights measured on day 0. The first treatment was administered orally approximately 30 minutes prior to surgery or at the same time the preanesthetic agents were given. Subsequent administrations were given at approximately the same time each day on days 1 and 2.

### 2.5. Randomization and Blinding Procedures

Cats were randomly allocated to two treatment groups in blocks of three, in a 2 : 1 ratio of robenacoxib : placebo. Separate computer-generated randomization schedules were prepared for each investigator's site. Blinding was maintained using robenacoxib and placebo tablets, and blister packaging, of identical appearance, as well as having a “Dispenser” identified at each clinic responsible for dispensation and reconciliation of used and unused test material. There were no reports of accidental unblinding at any site.

### 2.6. Clinical Examinations

Clinical examinations and body weight measurements were made prior to enrollment, on day 0 and day 2, in cases of early withdrawal and for any animal which experienced a serious adverse event (AE). Animals receiving rescue analgesia were monitored for at least 24 hours after intervention. Owners of all study cats received a follow-up call 3–7 days after hospital discharge.

### 2.7. Efficacy and Tolerability Assessment

For efficacy, treatment groups were compared on a success/failure basis as the primary efficacy variable, with treatment failure defined as the need for rescue therapy to control postoperative pain. The following secondary efficacy variables were assessed: posture, behavior (viewed from a distance and following social interaction), pain elicited on palpation (paws and soft tissue incision site), and overall pain control using numerical rating scales (see Table 6). A baseline evaluation of secondary efficacy variables was made on day 0 after the cat was acclimated for a minimum of 2 hours and prior to administration of tablets or the preanesthetic.

Evaluations of efficacy variables and the need for “rescue therapy” were conducted at 0 and 30 minutes (±10 minutes); 1 hour (±10 minutes); 3, 5, and 8 hours (±15 minutes); 24, 28, 32, 48, and 52 hours (±1 hour) after extubation. Each case was monitored at all time points by a single veterinarian.

Tolerability was assessed from reported AEs, pre- and postsurgery clinical pathology variables (hematology, clinical chemistry, and urinalysis), and body weight change.

### 2.8. Statistics

All analyses were performed using SAS/STAT software (Version 9.1.3, SAS Institute Inc., Cary, NC, USA). Summary statistics were performed for all variables. All statistical tests were evaluated at a 2-sided 0.05 level of significance. The study target was a minimum of 150 evaluable cases, 100 treated with robenacoxib and 50 with placebo, with a minimum of 9 cats per center and a maximum of 40% of the total.

The primary efficacy variable was “rescue” with superiority established by a reduction in the proportion of cases rescued in the robenacoxib group compared to the placebo group. The general linear mixed model (PROC GLIMMIX) was used with “treatment” as a fixed effect and “site” and “treatment by site” as random effects. The endpoint was classified as success (not rescued) or failure (rescued). A logit link function was used. The time to rescue was assessed via survival analysis using a Kaplan-Meier plot and the log rank test (PROC LIFE TEST). The onychectomy procedures were compared across treatment groups using Fisher's Exact test (PROC FREQ).

For the secondary efficacy variables, the general linear mixed model included the fixed effects of “treatment”, “time”, their interaction, and the random effects of “site” and “treatment by site”. Last Observation Carried Forward (LOCF) was used for any animal requiring rescue therapy on the day of surgery [12]. Only data from the day of surgery (extubation to 8 hours) were analyzed statistically as the LOCF assumption, that the animal's response remains unchanged, becomes weaker the longer assessment times are from the time of surgery.

Hematology, serum chemistry, and urinalysis variables were evaluated using analysis of covariance (PROC MIXED) with the fixed effect of “treatment”, random effects of “site” and “treatment by site”, and the pretreatment value used as a covariate. Data were transformed (logarithmic, square root, or reciprocal) when appropriate to meet normality assumptions.

Body weight change from pretreatment to study exit was analyzed using an analysis of variance (PROC MIXED) with the fixed effect of “treatment” and random effects of “site” and “treatment by site”.

## 3. Results

### 3.1. Demographic and Baseline Data

Two hundred and forty-nine cats were enrolled from twelve geographically distant sites in the USA between April and September 2008; 167 cats received robenacoxib and 82 received placebo. Ages ranged from 5 months to 13 years 7 months, with 61% of cats 6 to 12 months old. One hundred and ten cats were male and 139 female. Body weights ranged from 2.5 to 7.4 kg with 86% of cats weighing 2.5 to 4.4 kg. Twelve breeds were represented with 79% domestic short hair. Data from all 249 cats were included in the safety assessments. Five cats were not included in the primary efficacy analyses for the following reasons: minimum recruitment target of nine cases per site not reached (3); surgery was not undertaken and wrong dosage of test article was administered (1); preassessment was not completed prior to surgery (1). Four additional cats were not included in the analysis of the secondary efficacy variables as they were withdrawn from the study due to an AE being not associated with pain. Therefore the primary efficacy variable was analyzed with data from 244 cats (164 robenacoxib and 80 placebo), while secondary efficacy variables were analyzed with data from 240 cats (161 robenacoxib and 79 placebo). All cats underwent forelimb onychectomy, and either castration (110 male, 73 received robenacoxib and 37 placebo) or ovariohysterectomy (139 female, 94 received robenacoxib and 45 placebo). The procedures for the onychectomy were guillotine-type nail trimmer in 101 cats (41.4%), laser scapel in 90 cats (36.9%), and surgical in 53 cats (21.7%), with no significant differences between the robenacoxib and placebo groups (*P* = 0.95). The mean (range) dosage of robenacoxib administered was 1.84 (1.03–2.40) mg/kg.

In addition to butorphanol and local bupivacaine, acepromazine, atropine, diazepam, glycopyrrolate, isoflurane, ketamine, propofol, thiopental, and tiletamine/zolazepam and the antibiotics amoxicillin, with or without clavulanate, cefazolin, cefovecin, enrofloxacin, and penicillin were used in some cats.

### 3.2. Primary Efficacy Variable (Rescue Therapy)

**Figure 1:**
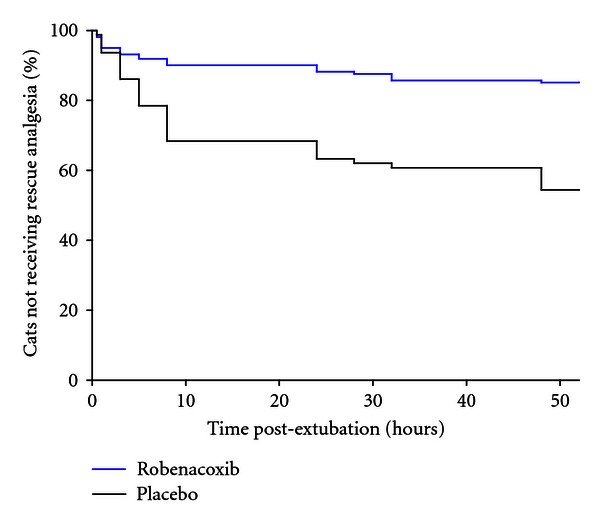
Kaplan-Meier plot of the percentage of cats not needing rescue analgesia after surgery. The risk of receiving rescue therapy was significantly (*P* < 0.0001) lower with robenacoxib.

Sixty-four animals received rescue analgesic therapy and were assessed as treatment failures (Table 1). A significantly (*P* = 0.048) lower proportion of rescues occurred in the robenacoxib group (16.5%) compared to the placebo (46.3%) with a slightly higher percentage in females (26.6%) compared to males (22.8%). The percentage of rescues was lower in the robenacoxib group for each onychectomy surgery subgroup, particularly the guillotine-type nail trimmer (Table 1). Survival analysis showed the risk of reaching treatment failure prior to study exit was significantly lower with robenacoxib (*P* < 0.0001; Figure 1).

### 3.3. Secondary Efficacy Variables

The treatment by time interaction was significant for posture, social interaction behavior, and palpation of the soft tissue incision site. There were significant differences in favor of robenacoxib in both posture (*P* < 0.05) and social interaction behavior (*P* < 0.05) at 3, 5, and 8 hours and in palpation of the soft tissue incision site (*P* < 0.01) at 5 and 8 hours (Table 2).

For behavior viewed from a distance, pain elicited on palpation, and overall pain control, the model did not converge; mean values were lower with robenacoxib compared to placebo. Mean (SD) values on the day of surgery in the robenacoxib and placebo groups were 1.10 (0.33) and 1.22 (0.45) for behavior viewed from a distance; 1.87 (1.01) and 2.10 (1.30) for pain elicited on palpation of the paw; 1.18 (0.47) and 1.40 (0.68) for overall pain control, respectively.

### 3.4. Tolerability

There were 35 AE reports in 27 of the 167 cats receiving robenacoxib (16.2%) and 16 reports in 12 of 82 cats receiving placebo (14.6%). There were no breed, age, or gender predilections for reported AEs.

AEs deemed clinically serious included cases that were moderate to severe in intensity and required medical intervention. There were 11 serious AEs reports for 7 cats treated with robenacoxib and 8 reports for 6 cats treated with placebo. All clinically serious AEs in the robenacoxib group recovered completely. A serious AE involving dyspnea, tachypnea, tachycardia, bradycardia, weakness, and incoordination in the placebo group resulted in the death of one cat. Necropsy results were suggestive of cardiomyopathy. The most frequently reported AEs included such clinical signs as surgical site bleeding, infected surgery sites, inappetance, and lethargy (Table 3). Of the 7 incision site bleeding cases in the robenacoxib group, 6 were limited to the declaw site, in most cases involved only 1-2 digits of one paw and did not include castration or ovariohysterectomy surgery sites.

Thirty-five cats (26 robenacoxib (15.6%) and 9 placebo (11.0%)) were reported with abnormal findings on post-study follow-up telephone contact. The majority of the findings comprised known sideeffects related to onychectomy, ovariohysterectomy, or castration, including pain at incision sites, infection, bleeding, and temporary-behavior-related changes (e.g., decreased appetite, lethargy, chewing at feet, hiding) possibly associated with residual surgery-induced pain.

Variables for clinical pathology (Tables 4 and 5) and body weight change (*P *= 0.16) were similar between groups.

## 4. Discussion

Administration of robenacoxib tablets approximately 30 minutes prior to surgery and then once daily for two subsequent days was well tolerated and provided better control than placebo of postoperative pain and inflammation in cats undergoing forelimb onychectomy in combination with an ovariohysterectomy or castration. The proportion of cats needing rescue analgesia was significantly lower in the robenacoxib group compared to the placebo. Robenacoxib also demonstrated efficacy in soft tissue incision site pain, social interaction behavior and posture. Once daily dosing with robenacoxib provided good efficacy over the dosing interval. These results support previous findings that robenacoxib has a relatively long duration of action despite its short blood half life, explained by concentration and persistence at sites of inflammation [13].

Onychectomy is a standard model for testing analgesics in cats, either alone or in combination with neutering [8, 14–17]. Cases in this study had both orthopedic (onychectomy) and soft tissue (ovariohysterectomy or castration) components. Frequency of rescue analgesia was 16.5% with robenacoxib and 46.3% with placebo, used in addition to presurgery butorphanol and bupivacaine nerve blocks. Previous studies in cats undergoing onychectomy with or without neutering reported the following frequencies of rescue therapy: 95% (negative control) and 17% (pre- and postsurgery butorphanol) [8]; 67% and 71% with single presurgery meloxicam and butorphanol, respectively [16]; 27% (transdermal fentanyl) and 9% (butorphanol) [14]. In none of these studies did cats receive local anesthesia prior to surgery, although one study concluded that a four-point regional nerve block with bupivacaine, as used in our study, provided no benefit when added to buprenorphine [17]. In a fourth study, no rescue therapy was administered to cats undergoing only onychectomy receiving butorphanol or transdermal fentanyl [15].

The design of this study was a prospective, randomized, blinded comparison of robenacoxib to a placebo in a parallel-group design. Administration of placebo to animals in pain studies raises ethical and welfare issues; however, these were overcome by providing both butorphanol and regional bupivacaine nerve blocks to all cats prior to surgery.

The efficacy of butorphanol has been demonstrated previously compared to a negative control in cats undergoing onychectomy, with or without neutering, at an intramuscular dosage of 0.2 mg/kg, lower than the 0.4 mg/kg dosage used in this study [8]. Local nerve block with bupivacaine has also been evaluated in cats undergoing onychectomy, although in one study it was concluded to have no significant benefit when added to buprenorphine [17]. In addition, Investigators were instructed to administer additional analgesia immediately if judged necessary. Use of rescue therapy was similar in both groups immediately following extubation (Figure 1) suggesting that the combination of presurgical butorphanol and bupivacaine nerve blocks was effective postoperatively for approximately 3 hours. A 185-minute duration of action was reported for intramuscular administration of 0.4 mg/kg butorphanol using the thermal threshold method in healthy cats [18].

The frequency of reported AEs was similar in the robenacoxib (16.2%) and placebo (14.6%) groups. The most frequently reported AEs included surgical site bleeding, infected surgery sites, inappetance, and lethargy, complications typically associated with surgery. Reported incision site bleeding was restricted to the onychectomy site and is consistent with the report that 50% of onychectomy surgeries have complications including pain, bleeding, and lameness, regardless of onychectomy technique [19, 20]. At supratherapeutic (5 or 10 mg/kg) dosages daily for 28 days, robenacoxib was well tolerated and had no detectable effect on the partial activated prothromboplastin time or hematology variables in healthy young cats [7]. There was no evidence of any AEs of robenacoxib on target organs most sensitive to NSAID toxicity (gastrointestinal tract, kidney, and liver), consistent with previous studies in healthy cats [4, 7].

The principal limitation of the study included the use of scoring schemes that have not been validated. Initial validation of another evaluation scheme in cats was reported in 2011 [21] but was not available for this study. In addition, the need for rescue therapy was based on a global subjective evaluation of the cat by the veterinarian. However, statistical superiority was shown for robenacoxib for the primary and some secondary efficacy endpoints, indicating that the study design and statistical power were sufficient to meet the study objectives.

The study was placebo-controlled and therefore provides the highest evidence for the efficacy and tolerability of robenacoxib. The lack of a positive control does not allow direct comparison with reference products. As noted previously, the frequency of rescue therapy with robenacoxib in this study (16.5%) differs from values reported with meloxicam (67%) [16]. However, results between the two studies cannot be compared directly due to different methods, for example, lack of nerve blocks and presence of clear criteria for use of rescue therapy in the Carroll et al., study [16].

Analysis of the secondary efficacy endpoints, clinical signs of pain and inflammation, was a challenge due to the unequal frequency of withdrawal of cases after administration of rescue therapy in the two groups. The data are therefore presented both without modification (which should lead to a bias in favor of the placebo group) and with LOCF analysis (which may also lead to biases, including possibly in favor of robenacoxib). The LOCF methods have limitations [12] but were justified in this study since they were used only in cases proactively withdrawn with insufficient efficacy, and only for a limited period (up to 8 hours postextubation).

In conclusion, robenacoxib was effective and well tolerated, when used in combination with presurgical buprenorphine and a forelimb bupivacaine ring block, in the control of postoperative pain and inflammation in cats undergoing onychectomy plus ovariohysterectomy or castration.

## Figures and Tables

**Table 1:** Primary efficacy variable: number of cats (percentages) receiving additional analgesia (rescued, treatment failure) according to the surgical method used for onychectomy.

Treatment	Surgery type	Failure (rescued)	Success (not rescued)
	Guillotine-type nail trimmer	9 (13.0%)	60 (87.0%)
Robenacoxib	Laser scalpel	10 (16.7%)	50 (83.3%)
	Surgical	8 (22.9%)	27 (77.1%)

	Total	27 (16.5%)	137 (83.5%)

	Guillotine-type nail trimmer	20 (62.5%)	12 (37.5%)
Placebo	Laser scalpel	9 (30.0%)	21 (70.0%)
	Surgical	8 (44.4%)	10 (55.6%)

	Total	37 (46.3%)	43 (53.7%)

**Table 2:** Summary statistics for secondary efficacy variables. On day 0, LOCF was applied. The treatment by time interaction was significant for all variables, therefore comparisons at each time (0.5, 1, 3, 5, and 8 hours) are reported. On days 1 and 2, data are provided without LOCF applied. Groups were not compared statistically after 8 hours.

Treatment	Time (hours)	*n*	Posture score			Social interaction behavior score			Soft tissue incision site pain score		
			Mean	SD	*P* value	Mean	SD	*P* value	Mean	SD	*P* value
Summary statistics with LOCF											

Robenacoxib	0	161	1.15	0.65	0.9156	1.16	0.66	0.7519	1.22	0.42	0.1092
Placebo		79	1.14	0.59		1.13	0.59		1.23	0.42	
Robenacoxib	0.5	160	1.26	0.61	0.9730	1.26	0.60	0.8598	1.31	0.52	0.6441
Placebo		79	1.29	0.64		1.29	0.60		1.38	0.58	
Robenacoxib	1	161	1.34	0.66	0.4257	1.36	0.69	0.4602	1.39	0.58	0.3371
Placebo		79	1.43	0.67		1.46	0.73		1.56	0.78	
Robenacoxib	3	161	1.31	0.61	0.0039	1.34	0.66	0.0373	1.43	0.61	0.1095
Placebo		79	1.61	0.74		1.58	0.78		1.65	0.85	
Robenacoxib	5	161	1.35	0.66	0.0002	1.37	0.68	0.0019	1.42	0.61	0.0031
Placebo		79	1.76	0.8		1.73	0.83		1.75	0.90	
Robenacoxib	8	161	1.40	0.64	0.0004	1.36	0.67	0.0005	1.49	0.63	0.0087
Placebo		79	1.82	0.84		1.78	0.90		1.78	0.90	

Summary statistics without LOCF											

Robenacoxib	24	145	1.37	0.55	ND	1.23	0.48	ND	1.43	0.59	ND
Placebo		54	1.52	0.72		1.30	0.50		1.44	0.72	
Robenacoxib	28	142	1.29	0.47	ND	1.17	0.43	ND	1.40	0.55	ND
Placebo		50	1.38	0.53		1.34	0.59		1.36	0.66	
Robenacoxib	32	141	1.33	0.55	ND	1.17	0.45	ND	1.43	0.56	ND
Placebo		49	1.41	0.54		1.33	0.47		1.31	0.65	
Robenacoxib	48	138	1.25	0.47	ND	1.13	0.40	ND	1.39	0.53	ND
Placebo		48	1.42	0.58		1.27	0.61		1.27	0.61	
Robenacoxib	52	137	1.27	0.46	ND	1.09	0.32	ND	1.36	0.51	ND
Placebo		43	1.33	0.47		1.16	0.37		1.14	0.41	

ND: not determined.

**Table 3:** Frequency of reported adverse events (number of cats).

Adverse event	Robenacoxib *n* = 167 cats	Placebo *n* = 82 cats
Incision site bleeding	7	1
Incision site infection	6	2
Inappetance, weight loss	4	2
Decreased activity, lethargy, hiding	4	1
Vomiting	4	1
Bloody stool, diarrhea	3	1
Cystitis/hematuria	3	0
Hair loss, excoriation, bruising	2	0
Incoordination, weakness	1	1
Respiratory, cardiac arrest	1	0
Death	0	1

*Cats may have experienced more than one type or occurrence of an adverse event during the study.

**Table 4:** Selected hepatic biomarkers and hematological variables measured at study exit.

	Robenacoxib *n* = 150				Placebo *n* = 73				Reference range	*P* value
Variable: units	Mean	SD	# Cases^∗^		Mean	SD	# Cases^∗^			
			High	Low			High	Low		
Alkaline phosphatase (ALP): U/L	37.19^∗∗^	26.71	0	1	46.38	42.89	2	0	6–102	0.93
Alanine aminotransferase (ALT): U/L	53.14	22.13	4	0	60.03	52.44	4	0	10–100	0.30
Aspartate aminotransferase (AST): U/L	35.03	25.25	5	0	35.48	25.73	2	0	10–100	1.0
Bilirubin: mg/dL	0.18	0.07	1	0	0.18	0.09	2	0	0.1–0.4	0.76
Hematocrit: %	37.51	5.47	5	3	37.07	5.64	0	5	29–48	0.18
Hemoglobin: gm/dL	12.04	1.61	2	4	11.84	1.64	0	2	9.3–15.9	0.12
Platelet Count^∗∗∗^: 10^3^/*μ*L	290.15	107.77	3	21	306.16	105.16	4	5	200–500	0.45
Red Blood Cell Count: 10^6^/*μ*L	8.43	1.19	8	0	8.12	1.30	2	2	5.92–9.93	0.054

*Number of cases with values higher (high) or lower (low) than reference range.

**ALP (*n* = 149).

***Platelet clumps were noted in several cases preventing precise determination of count and falsely decreasing the platelet number.

**Table 5:** Selected serum and urine renal biomarkers measured at study exit.

Variable: units	Robenacoxib *n* = 150				Placebo *n* = 73				Reference range	*P* value
	Mean	SD	# Cases^∗^		Mean	SD	# Cases^∗^			
			High	Low			High	Low		
Urea Nitrogen: mg/dL	23.21	4.89	1	1	22.33	5.86	0	4	14–36	0.48
Creatinine: mg/dL	1.07	0.24	0	1	1.06	0.26	0	0	0.6–2.4	0.52
BUN : Creatinine Ratio	22.74	7.07	5	0	21.74	6.06	2	0	4–33	0.87
Total Protein: g/dL	7.01	0.61	0	1	7.11	0.63	0	0	5.2–8.8	0.60
Albumin: g/dL	3.21	0.32	0	4	3.15	0.29	0	2	2.5–3.9	0.14

	Robenacoxib *n* = 133				Placebo *n* = 65					
Variable: units	Mean	SD	# Cases^∗^		Mean	SD	# Cases^∗^		Reference range	*P* value
			High	Low			High	Low		

Urine specific gravity	1.060	0.01	32	1	1.050	0.02	10	0	1.015–1.060	0.020^∗∗^

*Number of cases with values higher (high) or lower (low) than reference range.

**P  value < 0.05.

**Table 6:** Summary of secondary efficacy variables.

Variable	Description	Scale
Posture	Cat's mobility and posture within the cage	(1) Normal
		(2) Mildly abnormal
		(3) Moderately abnormal
		(4) Severely abnormal

Behavior	Cat's overall comfort, levels of aggression and vocalization, and ease of handling	Behavior as viewed from a distance
		(1) Appears comfortable
		(2) Questionable comfort
		(3) Distressed animal
		Behavior following social interaction
		(1) Normal
		(2) Mildly abnormal
		(3) Moderately abnormal
		(4) Severely abnormal

Pain on palpation	Amount of pressure applied to sites adjacent to incisions that elicited a pain response (e.g., withdrawal of paw, discomfort, or vocalization).	Paw onychectomy. Pressure assessed using a Palpometer^*∗*^. Response based on audio feedback:
		(1) 5 beeps (greatest recorded pressure)
		(2) 4 beeps
		(3) 3 beeps
		(4) 2 beeps
		(5) 1 beep (lightest recorded pressure)
		Castration or ovariohysterectomy skin incision. Based on subjective evaluation:
		(1) Significant pressure
		(2) Moderate pressure
		(3) Slight pressure

Overall pain control	Subjective overall assessment	(1) Well controlled
		(2) Moderately controlled
		(3) Poorly controlled

*The index finger mounted device (Palpometer, University of Victoria Innovation and Development Corp. Victoria, BC, Canada) scores of 1, 2, 3, 4, or 5 beeps corresponded to pressures of 200, 300, 450, 600, and 800 gf/cm^2^, respectively [22]. All devices were calibrated before use.
